# Identification of mouse gaits using a novel force-sensing exercise wheel

**DOI:** 10.1152/japplphysiol.01014.2014

**Published:** 2015-07-02

**Authors:** Benjamin J. H. Smith, Lottie Cullingford, James R. Usherwood

**Affiliations:** Structure and Motion Laboratory, Royal Veterinary College, Hertfordshire, United Kingdom

**Keywords:** gait, locomotion, biomechanics, ground reaction force, mouse

## Abstract

The gaits that animals use can provide information on neurological and musculoskeletal disorders, as well as the biomechanics of locomotion. Mice are a common research model in many fields; however, there is no consensus in the literature on how (and if) mouse gaits vary with speed. One of the challenges in studying mouse gaits is that mice tend to run intermittently on treadmills or overground; this paper attempts to overcome this issue with a novel exercise wheel that measures vertical ground reaction forces. Unlike previous instrumented wheels, this wheel is able to measure forces continuously and can therefore record data from consecutive strides. By concatenating the maximum limb force at each time point, a force trace can be constructed to quantify and identify gaits. The wheel was three dimensionally printed, allowing the design to be shared with other researchers. The kinematic parameters measured by the wheel were evaluated using high-speed video. Gaits were classified using a metric called “3S” (stride signal symmetry), which quantifies the half wave symmetry of the force trace peaks. Although mice are capable of using both symmetric and asymmetric gaits throughout their speed range, the continuum of gaits can be divided into regions based on the frequency of symmetric and asymmetric gaits; these divisions are further supported by the fact that mice run less frequently at speeds near the boundaries between regions. The boundary speeds correspond to gait transition speeds predicted by the hypothesis that mice move in a dynamically similar fashion to other legged animals.

most animals exhibit changes in gait as their speed changes, most commonly (in quadrupeds) from walking to trotting, and trotting to galloping, as they accelerate. These gait transitions often occur at specific speeds that scale with animal size, suggesting that the dynamics of legged locomotion are similar across many terrestrial vertebrates (2, 16, 36). As well as providing valuable information about the fundamental dynamics of locomotion, measurements of gait parameters are increasingly used in the detection and treatment of neuromuscular disorders (8, 15). Mice are one of the most commonly used animals for this type of research, since they are easy to maintain and breed, and there are numerous inbred and genetically modified strains available that display a variety of physical and behavioural characteristics. The literature on mouse locomotion suggests that in mice the link between gait and speed is much less clearly defined than in other, mostly much larger, animals. Some studies have claimed that mice use only walking and trotting gaits throughout their entire speed range (10), while others have observed a transition from trotting to galloping at a repeatable speed (16, 34), which follows the scaling relationships found for other animals. A comprehensive study of mouse gaits at a range of speeds found that mice used symmetric gaits in 22.9% of samples and nonsymmetric gaits in 77.1% of samples (17). The speed ranges of the symmetric and nonsymmetric gaits were similar, with the symmetric gaits occurring at velocities between 0.20 and 0.8 5 m/s, and the nonsymmetric gaits occurring at velocities between 0.09 and 0.88 m/s; however, no specific break points between gaits could be identified, and therefore, it was concluded that the formulas used to calculate gait transition speeds did not apply in mice.

One commonly used technique for studying gait is to measure the ground reaction forces (GRF) between the animal's feet and the substrate. However, most commercially available force plates are intended for use with humans and other large animals and are not suitable for use with smaller animals such as mice. Many researchers who wish to study rodent biomechanics (particularly for medical research) have opted to use systems such as Digigait or CatWalk, which use cameras to identify contacts between the paws and the substrate and hence measure kinematic variables such as stance and stride periods and stride lengths (29, 39). Although GRF can be estimated from these parameters, it is sometimes desirable to measure GRF directly; Zumwalt (41) opted to modify an existing plate to measure the GRF exerted by mice running overground, while others have built their own plates by placing load cells under a walkway (9, 13, 28). However, mice tend to run intermittently overground and on treadmills and often require external stimulation to begin running; this can make collecting datasets of repeated strides extremely time consuming. In contrast, mice will voluntarily use exercise wheels both in the laboratory (3) and in the wild (30), running for around 5 to 6 h total per day in the laboratory, and achieving speeds of more than 1.1 m/s (3, 12). Assuming an average stride frequency of 6–7 Hz, this translates to more than 100,000 strides per day. Large amounts of data can therefore be collected autonomously, with negligible disturbance or deviation from standard husbandry. This may be particularly useful in pain studies, where the presence of humans while measurements are being made may suppress responses (35).

Roach et al. (33) describe a commercial wheel modified to measure mouse GRF by removing one rung and replacing it by a rung suspended between two strain sensitive brackets which measured force in the normal and tangential directions (equivalent to vertical and horizontal GRF). Although this system was successfully used to collect force data from mice running in the wheel, it had a number of limitations: firstly, only one of the rungs was able to collect force data, making it more difficult to collect data from multiple feet for a given stride or to repeatedly collect data from the same foot over consecutive strides. Secondly, the use of strain gauges to measure force resulted in an error caused by gravitational forces on the sensors as the wheel rotates. The construction of strain gauges makes them particularly susceptible to electromagnetic noise due to parasitic capacitative coupling between the parallel tracks (37), as well as changes in temperature causing the sensor to expand or contract and therefore register a spurious signal (38). Strain gauges also require the use of a slip ring (see materials and methods for further discussion).

## MATERIALS AND METHODS

The first part of this section describes the construction and characterization of a rodent exercise wheel with integrated force sensors, while the second part describes an experiment using the wheel to investigate mouse gaits.

### Wheel Implementation

#### Design and construction.

While most previous studies of wheel running have used a traditional upright wheel (e.g., Refs. 3, 4), the wheel design presented here is based on the “saucer” wheels commonly sold in pet shops and increasingly used for environmental enrichment in scientific mouse colonies. Saucer wheels have a solid surface rather than bars, which is preferred by mice (4) and minimizes the risk of the animal being injured by slipping or catching a toe or tail in the mesh. The angled configuration also allows for a large wheel diameter (which mice also prefer; Ref. 4) while still keeping the vertical profile low enough to fit into a standard mouse cage. One disadvantage of this type of wheel is that the mouse is constantly running around a bend and on a slight incline, which could affect kinematic factors such as stance periods. The radius of the wheel discussed here was therefore made as large as possible [given the build size constraints of the available 3-dimensional (3D) printer] to minimize the effect of running around a curve, and the angle of the wheel was selected so that a mouse running at the front midpoint would be running on as level a surface as possible.

**Fig. 1.**
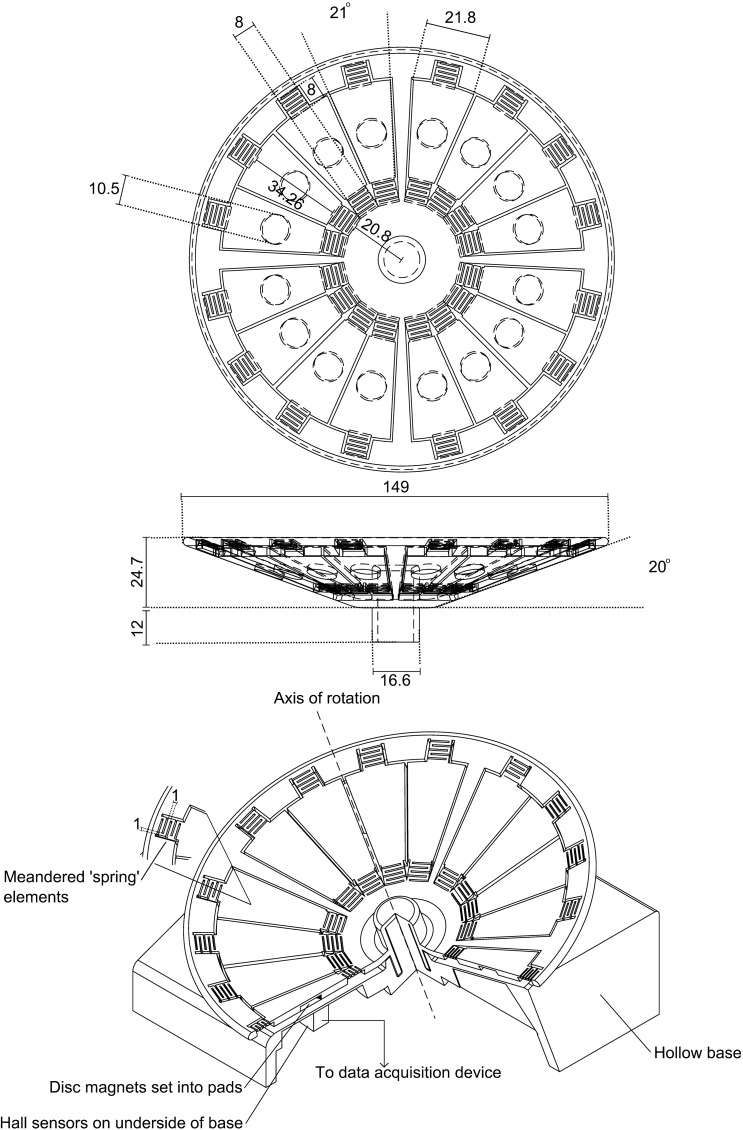
Cut away diagram of wheel. All dimensions are in mm. An array of 9 Hall sensors is mounted in the base; deflections of the pads produce changes in Hall sensor voltage that correspond to vertical ground reaction forces (GRF). All sensors are sampled simultaneously in parallel at 3 kHz to allow force measurement of multiple foot contacts. Data collection is triggered automatically when the wheel starts to rotate; data are saved in 5-s blocks until rotation stops.

The wheel is made up of a frame and 16 pads, arranged into four groups of 4 (Fig. 1). Each of these pads is attached to the frame by meandered elements that act as springs. On the underside of each pad is a socket for a magnet. The base is hollow to allow electronics to be mounted inside. Both the wheel and base were printed in Polyactide (PLA) using a Replicator 2 Desktop 3D printer (MakerBot Industries, Brooklyn, NY). PLA is a bioplastic derived from plant starch; it is nontoxic, presenting no risk to the mice if they ingest it. In more than 1,000 h of exposure to mice, minimal damage has occurred due to gnawing. Support material remaining after 3D printing was removed using a rotary tool and a scalpel. Magnets (10 mm diameter, 1.5 mm height, N42 Neodymium available at http://www.first4magnets.com/circular-disc-rod-magnets-c34/10mm-dia-x-1-5mm-thick-n42-neodymium-magnet-1kg-pull-p3632) were superglued into the sockets on each pad. The .stl files for the wheel and base, and a .gerber file for the Printed Circuit Board (PCB) layout is available on our website: http://www.rvc.ac.uk/Media/Default/staff/files/bsmith-wheel-files.zip.

An array of nine A1301ELHLT-T Hall effect sensors (Allegro Microsystems, Worcester, MA) were soldered onto the PCB and glued to the wheel base. Each sensor was positioned to align with the path of the centers of the magnets. When a pad is deflected downwards, the distance between the magnet and the sensor is reduced, and thus the strength of the magnetic field experienced by the Hall sensor directly below the pad is increased. The output voltage of the Hall sensor increases proportionally to the increase in magnetic field strength, and therefore; the force applied to the pad can be calculated from the voltage output from the Hall sensor. Throughout a given limb's stance phase the foot exerts a varying force on a pad; however, over the course of this stance phase the pad will pass over a number of adjacent Hall sensors as the wheel rotates. For a typical half-sine running signal the force measured by consecutive sensors will therefore increase until mid-stance, after which the force measured by consecutive sensors will decrease back to zero. A complete force trace for a stance is therefore produced by concatenating the samples measured by these adjacent sensors by selecting the maximum measurement at each time point for inclusion in the force trace. The GRF trace is therefore made up of single limb signals, rather than whole body forces; however, if multiple legs are producing similar forces, then two or more stances may be merged together; this typically occurs at the beginning and end of the stance phases when forces are lowest.

One advantage of using Hall sensors is that no physical or electrical contacts are required between the sensors and the wheel; this means that no slip ring is required, avoiding the associated noise, turning resistance, and cost. The sensors were connected to an NI USB-6218 DAQ (Data Acquisition) device (National Instruments, Austin, TX), which also provided them with 5-V DC power. Data were sampled simultaneously from all nine sensors in parallel at a rate of 3 kHz, using Labview SignalExpress 2009 (National Instruments). Data collection was triggered when the signal measured by the central sensor increased or decreased by 0.2 V; this is well above the noise threshold of 0.04 V and indicates that the wheel has begun to rotate. Data were saved in 5-s blocks to limit the impact of a power failure or system crash, which could corrupt the file currently being recorded; after saving the trigger was reset ready for the next wheel movement.

#### Calibration and characterization.

Calibration was carried out while the wheel was rotating, based on the mean values when a range of masses was applied. This was because it is not possible to know exactly which pad is over which sensor while a mouse is running on it. Identical masses were attached to each pad using Blu-tack (the mass of the Blu-tack was taken into account). The wheel was then rotated for 5 s, with readings taken from all sensors simultaneously at a rate of 3 kHz. A relationship between force and mean voltage measured by each sensor was then determined to produce a matrix of coefficients, which could be used to convert between voltage and force.

The effect of speed was measured by placing masses on each pad and rotating the wheel by hand at a range of speeds; this was repeated both unloaded and with masses of 4.9, 10.6, and 29.8 g (see appendix b). Significant effects of speed were observed in no sensors in the unloaded case, four sensors in the 4.9-g load case, one sensor in the 10.6-g load case, and two sensors in the 29.8-g load case. Only one sensor detected a significant effect in more than one case (the 10.6- and 29.8-g loads). This suggests that overall there is little systemic effect of speed on the voltage measured by the sensors.

The measurements for impulse response, linearity, and point of force application were made individually for each of the 16 pads on the wheel. Measurements were made while the wheel was stationary to minimize variation due to changes in the distance between the pad and the sensor, and to prevent vibrations other than the applied impulse. The impulse response was characterized for each pad by striking it with an aluminum rod and recording the vibrations. A fast Fourier transform (FFT) was then used to find the frequency with the strongest vibrations. The settling time was calculated as the time taken for the signal amplitude to return to within the noise limits. Table 1 lists the means and standard deviations of the impulse response characteristics of the pads.

**Table 1.** Unloaded wheel impulse response

Variable	
Natural frequency, Hz	94.3 ± 12.1
Settling time, s	0.06 ± 0.01
Max. Δ amplitude, V	0.01 ± 0.01
Phase at max. amplitude, s	0.04 ± 0.03

Values are means ± SD.

**Fig. 2.**
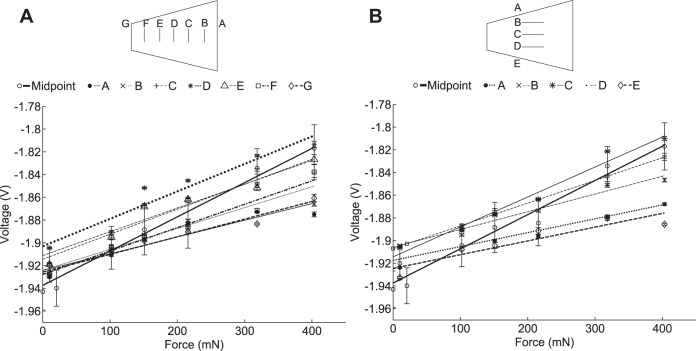
Linearity of wheel pads measured by placing calibration weights on the pads at a variety of positions. *A*: linearity measured across the pad from the outer to inner edge. *B*: linearity measured across the pad from the right to left edge. Voltage varies linearly with force (*R*^2^ > 0.9) in all cases.

Linearity for each pad was measured by placing calibration masses of increasing size on each pad. The masses ranged from 1 to 41 g or 3 to 137% body mass of a typical FVB mouse. The effect of the position of the calibration mass was also investigated; measurements were taken with the mass at the center of the pad for all the pads on the wheel, and at regular intervals from the outer to the inner edge and from the left to the right edge for a single pad. Figure 2 shows these values: Fig. 2*A* shows the variation in linearity as the point of contact moves from the outer to the inner edge of the pad, while Fig. 2*B* shows the variation as the point of contact moved from the left to the right of the pad. In each case voltage varies linearly with force (*R*^2^ > 0.9). The central point measures the highest force in both Fig. 2, *A* and *B*, with measured force values dropping off as the foot fall position gets further from the central point. Although there is some variation between the mean values for the different positions, the measurements for the outer and inner points are within one standard deviation of the middle point. One goal of this system is to collect data on mouse locomotion kinematics and kinetics without requiring a high-speed camera. Since without a camera it is not possible to determine the exact point of contact between a mouse's foot and a pad, we have instead attempted to reduce the effect of foot position on measured force. This is achieved firstly by designing the hardware to reduce moments due to off centre foot contacts; the width of the pads is fairly small relative to a mouse (hind) footprint, while the spring elements take up as much room on the sides of the pad as possible within the geometric constraints of the design. Secondly, the force reconstruction algorithm uses the highest force measurement at each sample interval. It is therefore more likely that measurements from footfalls near or at the centre of the pad are included in the force signal, so in large datasets there will only be minimal impact from foot position, as very little of the force signal will be comprised of footfalls on the edges of the pad.

#### Drift.

Drift was measured by sampling each of the sensors at 1-s intervals overnight (in total a period of 15.7 h). The mean change in voltage per hour over this time was 0.63 ± 0.10 mV (see appendix a for more detail). While this drift is negligible for the period over which the experiments reported here were carried out, if the wheel is to be used continuously over a longer period of time, regular adjustment of the zero points of the sensors might be desirable.

### Automated Recording and Classification of Gaits Using the Wheel

#### Animal experiments.

All observations were carried out in accordance with the United Kingdom Home Office recommendations for animal experiments. The subjects were five adult female FVB mice (age 4 mo, mass 34.3 ± 4.29g), which were housed in a temperature controlled room in the Royal Veterinary College Biological Services Unit. The usual 12-h light-dark cycle was maintained, and the mice were allowed food and water ad libitum. The mice had access to an exercise wheel similar to the one used in this study in their home cage and were acclimatized runners. Mice were placed in pairs in an arena with the wheel and allowed to explore and use the wheel voluntarily. A high-speed AOS X-PRI Mono camera (AOS Technologies) was placed in the arena pointing horizontally at the front midpoint of the wheel. A clear Perspex barrier was used to prevent the mice coming in contact with the camera or cables. When a mouse started running, the observer would start recording high-speed video of the wheel at 250 Hz.

#### Data processing.

**Fig. 3.**
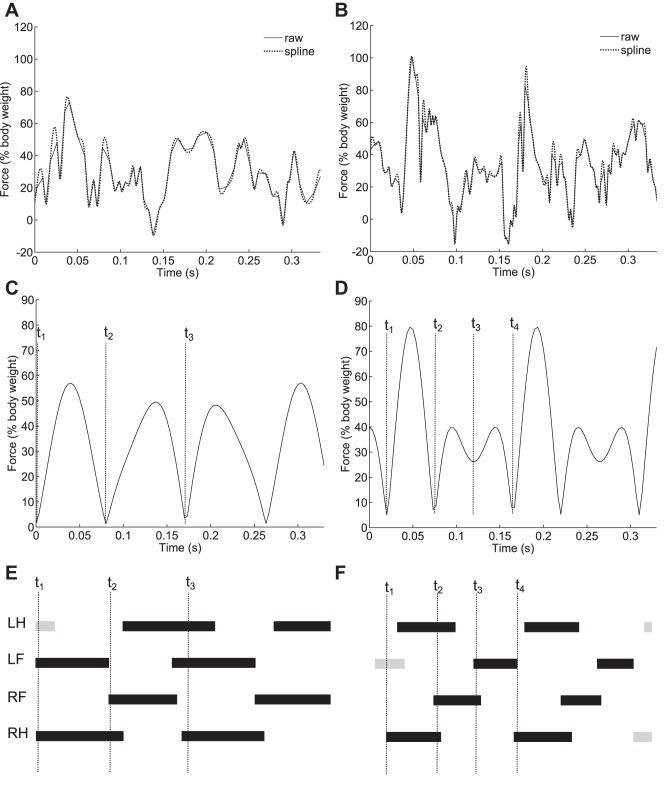
Reconstructing the gait from raw force data. *A*, *C*, and *E*: relate to a trotting gait. *B*, *D*, and *F*: relate to a half-bounding gait. *A* and *B*: raw forces and the smoothed signal. *C* and *D*: outputs of the inverse fast Fourier transform (IFFT). *E* and *F*: Hildebrand style gait diagrams that relate to the reconstructed signals in *C* and *D* (with dimensions taken from high-speed video). T_1_, T_2_, T_3_, and T_4_ illustrate how the reconstructed force signals and high-speed video were compared; mean stance times were compared with t_2_–t_1_, and mean stride periods were compared with t3-t1 (t4-t1 in the case of half bounding).

Figure 3 illustrates the gait reconstruction process. A custom MATLAB script (available from our website: http://www.rvc.ac.uk/Media/Default/staff/files/bsmith-wheel-files.zip) was used to calculate speeds and forces from the voltage signals from the Hall sensors and combine the signals into a single trace: speed was calculated from the voltage signal of the central sensor; since there is no magnet on the wheel's support struts there is a minimum in the voltage signal every time a strut passes over the sensor. Speed of the wheel (in revolutions per second) is calculated using the formula ω = *f*/4*s* where *f* is the sample frequency (here 3 kHz) and *s* is the number of samples between peaks. Force is calculated by first scaling the voltage signals using the calibration values and then by concatenating the maximum values at each time point. This means that if two legs are in contact at the same time only the greater exerted force is included in the force signal; the force signal is therefore comprised of the maximum force values over time, rather than the sum of force values (except in the case where both feet hit a single pad simultaneously, when the measured force is the summed force of the 2 feet). Finally, the signal was smoothed by interpolating with a cubic spline, using the MATLAB “spline” function (29a). To extract periodic gait parameters, the raw force data were first passed through an FFT. The two highest peaks of the FFT (neglecting the DC component) were identified. The rest of the FFT output was set to 0 (taking into account the fact that in the frequency domain the signal should be symmetric about *f*_s_/2, where *f*_s_ is the sample frequency; Ref. 32), and the heights of the two peaks were scaled such that the energy of the signal remained the same, so that it would still satisfy Parseval's theorem (31). An inverse fast Fourier transform (IFFT) was then applied to the signal to convert it back to a GRF. The overall effect of this algorithm is to apply a very selective band pass filter at frequencies determined from the data itself to the GRF, allowing any periodic signals due to inconsistencies in wheel construction to be removed, as well as higher frequency vibrations. Kinematic data were extracted from the reconstructed signals: the signal was divided into strides by identifying repeating features, and each stride was segmented into stances by identifying points where the force dropped to zero or very close to zero. Stance periods were measured between the minima, stride periods were measured between the repeating features, and duty factors were calculated as stance periods divided by the relevant stride periods. Only stances with a single peak were used for comparison with the stance periods and duty factors calculated from the high-speed video to ensure as much as possible that only the stance period of a single leg was being measured.

Peak forces measured by the wheel were compared with peak forces predicted from duty factor using an equation developed by Alexander et al. (1). This equation models GRF as a half sine wave, so that the peak force is a function of body weight, stance period, and stride period. In large animals such as horses, a bias factor is usually included in this calculation to take into account the fact that a greater proportion of bodyweight is supported by the fore legs than by the hind (40). Typically the ratio is 60% of body weight supported by the fore legs and 40% supported by the hind legs. However, Clarke and Still (9) found no significant difference between the impulses produced by fore and hindlimbs in mice.

Fore and hind leg impulses were calculated from the reconstructed force traces. Traces produced by a half bounding gait (e.g., Fig. 3*B*) were used, as signals produced by fore and hindlimbs could be distinguished. Similarly to Ref. 9, very little difference between fore and hindlimb impulses was found, with hindlimb impulses being 96.5 ± 2.45% of forelimb impulses. A fore: hind bias ratio of 0.51:0.49 was therefore used to calculate the peak forces predicted from the high-speed video kinematics.

While in some animals, such as cheetahs, the proportion of body weight supported by the hindlimbs increases significantly with speed (23), this does not appear to be the case with mice; even though the support ratios presented here were calculated at the upper end of mouse speeds using half bounding strides, they are very similar to the ratios in Ref. 9 that were calculated at the lower end of mouse speeds, using walking and trotting strides. Roach et al. (33) also measured kinematic parameters and forces of mice running in wheels and compared their results to values predicted by duty factors (1). They found that the hind legs had a much greater proportion of body support than the fore legs (0.84:0.16); it is possible that the disc like shape of the wheel presented here allows the animal to run on a less curved surface than the upright wheel used in (33), and therefore, it produces results more similar to overground or treadmill running.

#### Gait analysis.

The kinematic parameters measured from the high-speed videos were used to manually classify the gaits in the videos corresponding to the force traces. The gaits were classified into four categories: creeping (an exploratory, fairly intermittent walking gait typically observed just as the mouse has climbed onto, or is about to climb off, the wheel), trotting, galloping, and half bounding. A true walk was not observed, probably because the inertia of the wheel makes it harder to maintain constant movement for long bouts at low speeds, but perhaps also reflecting a true unwillingness to adopt steady walking.

A gait was defined as trotting when the smallest difference in footfall timings occurred between diagonal pairs of legs and as galloping or half bounding when the smallest difference in footfall timings occurred between the fore or hind pair of legs. Half bounding was distinguished from galloping when the hind legs met and left the ground near simultaneously (i.e., a difference of <0.025 s). A gait was classified as creeping when the footfall timings between diagonal pairs, and between fore and hind pairs, were similar.

While the two types of gait in Fig. 3 can be distinguished by eye based on the number and relative height of the peaks, not all the reconstructed traces fall into these two categories; therefore, a quantifiable characterization metric based on the half wave symmetry of the peaks of the stride force traces was designed. This metric is referred to as 3S (stride signal symmetry). We also wanted to determine whether mice transition between gaits at specific speeds, or whether as Herbin et al. (17) suggest they use a continuum of gaits, and if so, we wanted to characterize this continuum. The algorithm therefore classifies gaits by assigning them continuous variables, rather than by assigning them to discrete gait categories. A further advantage of this approach compared with traditional gait categorization is that it can be independent of video data, which typically is time and computationally intensive to collect and analyze. A “square” trot (25) would have the highest 3S value, while a half bound would have the lowest 3S value. A theoretical “ideal” bounding gait, with synchronous hind contacts evenly spaced with synchronous forelimb contacts, would also fall within the same 3S category as trotting; however, this gait has not been observed in “wild-type” mice in the laboratory (10) (although mice may use a wider range of gaits in the wild). 3S was calculated as follows: the time between peaks in the gait signal was differentiated numerically to produce a triangle or sawtooth wave. The more skewed this wave was, the lower the 3S value. To compare the skewness of the wave, it was differentiated a second time, and then was tested for half wave symmetry by calculating the mean square error between the wave and a copy of the wave that had been half wave shifted. A gait such as a trot would result in a triangular wave with equal peaks and trough magnitudes and would therefore have high half wave symmetry and a correspondingly high 3S value. A gait such as a half bound would result in a sawtooth wave which would have low half wave symmetry and hence a low 3S value.

### Statistics

Statistical analyses were carried out using MATLAB. All values are reported as means ± SD, and 95% confidence intervals are plotted in grey in the figures. Paired sample *t*-tests were used to evaluate the differences between the kinematic data from the video and the wheel, in all cases statistical significance was set as *P* < 0.05.

## RESULTS

### Automated Recording and Classification of Gaits Using the Wheel

#### Kinematics and force measurements.

**Fig. 4.**
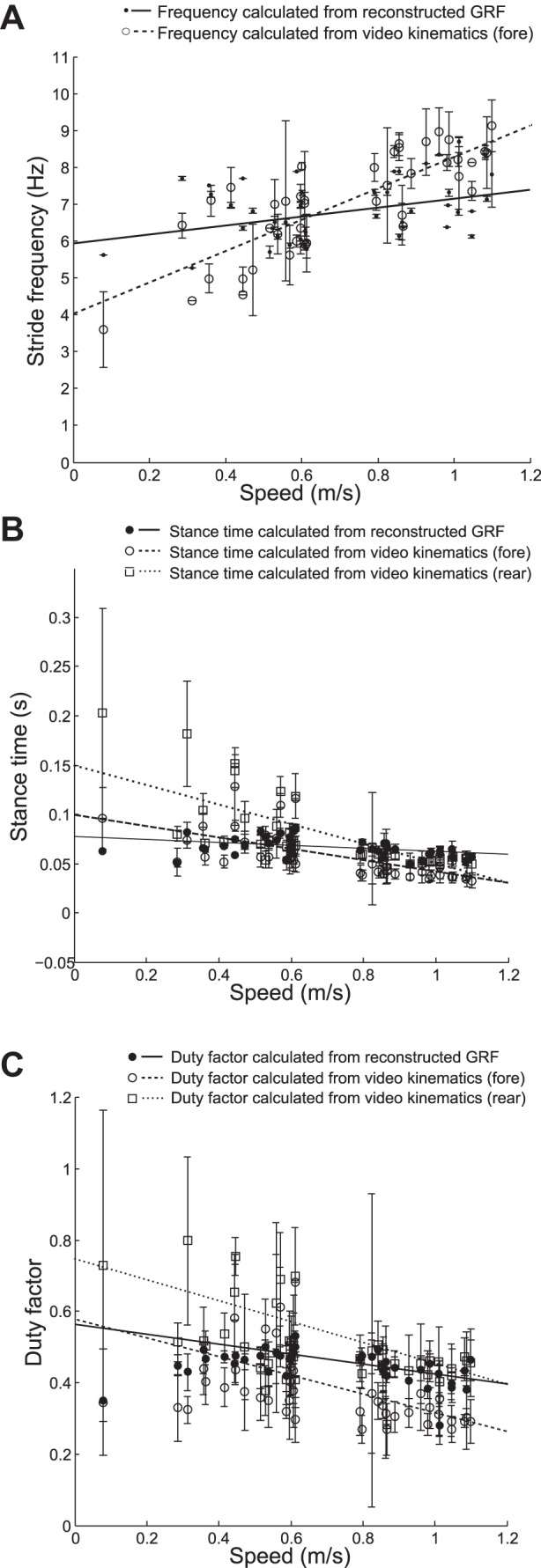
Comparisons of kinematic parameters between video and wheel data. *A*: stride frequencies vs. speed calculated using the reconstructed GRF signals and the video kinematics. There was a significant positive relationship with speed using both the reconstructed signals (*P* = 0.01) and the video kinematics (*P* < 0.001). *B*: stance periods vs. speed calculated using the reconstructed GRF signals and the video kinematics for fore and hind feet separately. There are significant negative relationships with speed for the stance periods calculated from the videos (fore: *P* < 0.001, hind: *P* < 0.001) and the stance periods calculated from the reconstructed signals (*P* = 0.008). *C*: duty factor vs. speed calculated using the reconstructed GRF signals and the video kinematics for fore and hind feet separately. Duty factor was calculated as stance period/stride period. There are significant negative relationships with speed for the duty factors calculated from the videos (fore: *P* = 0.003, hind: *P* < 0.001) and the duty factors calculated from the reconstructed signals (*P* = 0.007).

Stride frequency, duty factor, and stance periods were determined from high-speed video and used to provide an initial validation of the reconstructed force traces. The video and force data were synchronized based on their respective time stamps (the time stamps were both generated from the internal clock of the same PC) and segmented into blocks of three strides. Figure 4 shows means ± SD of kinematic parameters for each block, plotted against the mean speed of the block. Any block where the speed varied by more than 1 revolution per second (0.25 m/s) over the course of the block was discarded, so as to avoid accelerating and decelerating strides.

Figure 4*A* shows that stride frequency increases with speed when calculated from the video (*r* = 0.81, *P* < 0.01) and from the reconstructed GRF (*r* = 0.35, *P* = 0.024); however, the gradients of the lines of best fit are different. In particular, at speeds slower than 0.5 m/s the frequencies calculated from the video kinematics are lower than those calculated from the reconstructed signal, and at speeds higher than 1 m/s the frequencies calculated from the video kinematics are higher than those calculated from the reconstructed GRF. The standard deviations of the frequencies calculated from the video kinematics are much greater than those calculated from the reconstructed GRF; this is because the IFFT produces a periodic signal based on all three strides taken together, while the video kinematics are based on three unique strides. The root mean square (RMS) difference between the two sets of stride frequencies is 1.20 Hz. The stride frequencies calculated from the reconstructed signal range from 5.26 Hz (at a speed of 0.31 m/s) to 8.33 Hz (at a speed of 1.01 m/s). The stride frequencies calculated from the video range from 3.6 0 Hz (at a speed of 0.08 m/s) to 9.12 Hz (at a speed of 1.1 m/s). A paired sample *t*-test did not reject the hypothesis that the differences between the stride frequencies measured from the high-speed video and the wheel were drawn from a distribution with a mean of 0 (*P* = 0.30).

Figure 4*B* shows how the stance times of the fore and hind legs change with speed and how this compares to the reconstructed GRF. Both fore and hindlimb stance times decrease as speed increases (*r* = −0.69, *P* < 0.01 and *r* = −0.72, *P* < 0.001, respectively), although the hindlimb stance times reduce more quickly. As speed increases the fore and hindlimbs converge, becoming approximately the same around 1 m/s. The stance times calculated from the reconstructed signals also decrease as speed increases (*r* = −0.41, *P* = 0.008), however, at a different rate than fore and hindlimbs; at low speeds the reconstructed stance times are more similar to the forelimb stance times, while at higher speeds they become more similar to the hindlimb stance times (although as previously mentioned at speeds above 1 m/s both hind and forelimbs have similar stance times). As with the stride frequencies the video kinematic stance times have much higher standard deviations than the reconstructed stance times, again because the IFFT produces a periodic signal. The RMS errors between the reconstructed signal stance periods and the fore and hind leg stance periods are 0.024 and 0.035 s, respectively. Overall, fore legs had shorter stance periods than hind legs, with fore leg stance periods ranging between 0.13 s (at a speed of 0.45 m/s) to 0.03 s (at a speed of 1.1 m/s) and hind leg stance periods ranging between 0.20 s (at a speed of 0.08 m/s) and 0.04 s (at a speed of 0.87 m/s). Stance periods from the reconstructed signal ranged from 0.09 s (at a speed of 0.61 m/s) to 0.05 s (at a speed of 0.93 m/s). A paired sample *t*-test rejected the hypothesis that the differences between the fore leg stance times measured from the high-speed video and the wheel were drawn from a distribution with a mean of 0 (*P* = 0.03) but did not reject the hypothesis that the differences between the hind leg stance times measured from the high-speed video and the wheel were drawn from a distribution with a mean of 0 (*P* = 0.06). It also did not reject the hypothesis that the differences between the mean stance time of the fore and hind legs measured from the high-speed video and the wheel were drawn from a distribution with a mean of 0 (*P* = 0.98).

Figure 4*C* shows how duty factors for the fore and hindlimbs change with speed and how this compares to the duty factors calculated from the reconstructed GRF. Both fore and hindlimb duty factors decrease as speed increases (*r* = −0.68, *P* < 0.001 and *r* = −0.66, *P* < 0.001, respectively) and at similar rates. Duty factors for the hindlimbs are higher than those for forelimbs throughout the speed range, with forelimb duty factors ranging from 0.68 (at a speed of 0.61 m/s) to 0.27 (at a speed of 0.80 m/s) and hindlimb duty factors ranging from 0.80 (at a speed of 0.31 m/s) to 0.28 (at a speed of 0.87 m/s). Duty factors for the reconstructed signals also reduce as speed increases (*r* = −0.48, *P* = 0.002); similarly to the stance times at low speeds the duty factors are more similar to the forelimbs, and at higher speeds they are more similar to the hindlimbs. Duty factors for the reconstructed signals range from 0.53 (at a speed of 0.61 m/s) to 0.28 (at a speed of 1.01 m/s). The RMS errors between the reconstructed duty factors and the fore and hind leg duty factors are 0.11 and 0.12, respectively. Once again the data from the video kinematics has much higher standard deviations than the data from the reconstructed GRF, since the IFFT produces a periodic signal. A paired sample *t*-test rejected both the hypothesis that the differences between the duty factors measured from the high-speed video and the wheel were drawn from a distribution with a mean of 0 (*P* < 0.001), and the hypothesis that the differences between the duty factors measured from the high-speed video and the wheel were drawn from a distribution with a mean of 0 (*P* < 0.001). However, it did not reject the hypothesis that the differences between the mean duty factors measured from the high-speed video and the wheel were drawn from a distribution with a mean of 0 (*P* = 0.62).

**Fig. 5.**
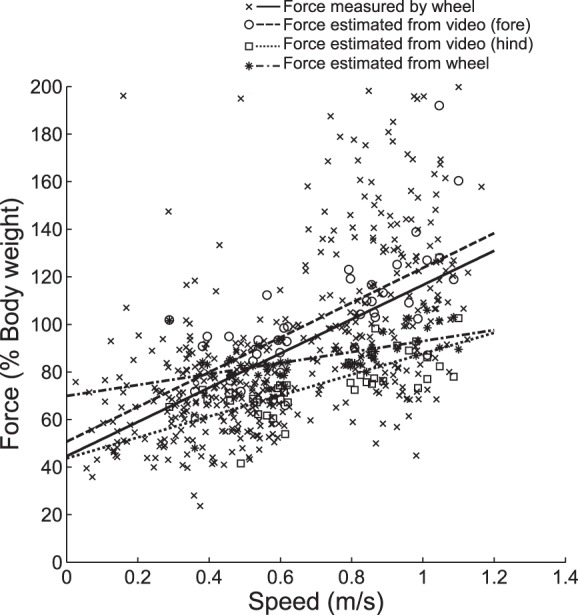
Peak limb forces measured by the wheel compared with peak forces predicted from duty factors. Comparing the impulses produced by fore and hindlimbs produced a fore: hindlimb bias of 0.51:0.49, this was used to calculate peak forces from duty factors using the equation found in Ref. 1. There are significant positive relationships between speed and the forces calculated from the video kinematics (*P* < 0.001) and those calculated from the reconstructed signals (*P* < 0.001).

Figure 5 compares the measured peaks forces with the forces estimated from the kinematic parameters, using the equation found in Ref. 1. As speed increases the forces estimated from the fore and hindlimb duty factors increase (*r* = 0.68, *P* < 0.001 and *r* = 0.70, *P* < 0.001, respectively), forces estimated from the wheel duty factors increase (*r* = 0.53, *P* < 0.001), and the forces measured by the wheel also increase (*r* = 0.58, *P* < 0.001). Force estimates are higher in the forelimbs than the hindlimbs throughout the speed range, ranging from 79.1% of body weight to 230.4% for the forelimbs and 33.2 to 102.4% for the hindlimbs. This reflects the fact that the duty factors were higher for the hindlimbs. Forces estimated from the wheel duty factors ranged from 48% body weight to 106%. The forces measured by the wheel range from 23.6% of body weight to 199.7%; however, the gradient of the change in force is more similar to that of the forelimbs than the hindlimbs. This could be because the forelimb forces were greater, so were more likely to comprise the peak forces.

## DISCUSSION

### Wheel Implementation

In this paper we have presented a novel design for a rodent exercise wheel that can measure GRF. A wheel combines advantages of both treadmill and overground running; large amounts of running data at a range of speeds can be collected quicker and more easily than with overground running, and mice are allowed to move voluntarily, reducing the amount of human interaction required, which might affect results. This also means that the wheel can be left in an animal's cage overnight to take advantage of the fact that mice are most active at night and to gather data on circadian rhythms. A previous study to design a force-sensing exercise wheel used an upright wheel with rungs, whereas we have used a solid horizontal wheel. This allows mice to run in a more natural posture.

The wheel is able to measure GRF and kinematic parameters without requiring a high-speed camera; this increases data throughput and reduces storage requirements. The wheel is able to detect trends in stride frequency, stance time, and duty factor associated with speed; however, it tends to underestimate times at low speeds (and hence overestimate stride frequencies and underestimate duty factors). This is most likely because it is not sensitive enough to detect the lower forces at slower speeds, so the beginning and end of stances where the forces are lowest get cut off. At higher speeds, the measured time intervals are more similar to those measured by the hind legs, which tend to have longer contact times than the fore legs; this leads to the underestimation of stride frequency at high speeds, stance times, and duty factors, most similar to the hind leg values.

Overall, the trends in stance time and duty factor tend to be most similar to the average of the fore and hindlimbs, rather than either the fore or hindlimbs; this is likely because at lower speeds the measured stance times and duty factors tend to be more similar to those of the fore legs and at intermediate and higher speeds they tend to be more similar to those of the hind legs. This also affects the forces estimated from the wheel duty factors; however, the slope of force data is very similar to that of the fore leg estimate, since the fore legs have a lower duty factor and therefore are more likely to be the source of the maximum force at a given sample time.

In the case where two feet hit a single pad simultaneously it is not possible to distinguish them. However, this mostly only occurs during the half bounding gait and only with the hind legs. It is therefore possible to determine whether a peak is from a front or hind leg based on the direction the wheel is rotating and the position of the sensor that reads the highest voltage; for example, if the wheel is rotating clockwise and signals are detected by Hall sensors 0 and 8 (i.e., the sensors on the right and left edges of the array, respectively), then the peak detected by sensor 0 must be due to a fore leg, and the peak detected by sensor 8 must be a hind leg.

### Comparison of Kinematic Parameters and Forces with Previous Studies

Previous studies have measured kinematic parameters of mouse gaits, some of these are summarized in Table 2. Although some of these studies only measured gait parameters across a small speed range, together they cover the range of speeds used by the mice in this article; 0.29–1.1 m/s. The substrate used does not appear to have much of an effect on stride frequency. There is fairly good agreement with the stride frequencies measured by this study with the overground results from Ref. 18; however, particularly at high speeds, the results measured with the wheel and from the video kinematics are lower than other studies. This could be because on our wheel the mice mostly used asymmetric gaits at higher speeds, as opposed to Ref. 17, where mice used both symmetric and asymmetric gaits equally throughout their speed range and Ref. 18 where the mice used the same gait throughout the speed range. Animals using asymmetric gaits tend to increase speed by increasing stride length, rather than by increasing stride frequency (16, 17), so stride frequencies would be lower than for symmetric gaits where stride frequency continues to increase with speed.

**Table 2.** Values from literature for mouse gait parameters

	Substrate	Speed Range, m/s	Stride Frequency, Hz	Stance Period, s	Duty Factor	Peak Vertical GRF, %body wt
Wheel measurement	Wheel	0.29–1.1	5.26 ± 0.0–8.70 ± 0.15	0.09 ± 0.0–0.05 ± 0.0	0.53 ± 0.02–0.28 ± 0.05	23.6–199.7
Video data	Wheel	0.29–1.1	3.59 ± 1.0–8.92 ± 0.7	(F) 0.13 ± 0.04–0.03 ± 0.01	(F) 0.68 ± 0.0–0.29 ± 0.06	N/A
				(H) 0.20 ± 0.11–0.05 ± 0.01	(H) 0.80 ± 0.24–0.28 ± 0.0	
Ref. 33	Wheel	0.28–0.60	N/A	0.108 ± 0.4	0.58 ± 0.01	(H) 90–115
Ref. 17	Treadmill	0.09–0.88	2.4–8.6	0.41–0.04	N/A	N/A
Ref. 18	Treadmill	0.08–1.01	1.8–11.4	0.44–0.03	0.83–0.25	N/A
Ref. 9	Overground	0.14–0.43	2.4–6.0	(F) 0.164 ± 0.01	(F) 0.62	(F) 63.5 ± 0.6
				(H) 0.19 ± 0.01	(H) 0.70	(H) 58.4 ± 0.7
Ref. 18)	Overground	0.20–1.30	3.2–10.4	0.21–0.03	0.83–0.25	N/A
Ref. 34	Overground	0.05–1.15	1.5–12.5	N/A	0.75–0.15	N/A

Values are means ± SD (where available).

F, forelimbs; R, hindlimbs; GRF, ground reaction forces.

On the other hand, at low speeds the values for stance time measured overground are much lower than those measured on treadmills, at higher speeds the overground stance times are higher. There is good agreement between the values measured on our wheel and the stance times from Refs. 9 and 18, particularly the hindlimb stance times. The other wheel study (33) only reports average stance time over a fairly low speed range; however, the reported values are much closer to the values measured here than values measured overground or on treadmills. Interestingly, although the value reported in Ref. 33 is for a hindlimb stance time, it is much closer to the forelimb stance time measured in this article. There is not much difference between duty factors measured overground, on the treadmill and on the wheel; our results also agree with these reported values.

These comparisons suggest that running on both the flat wheel and the upright wheel in Ref. 33 is more similar to running overground than running on a treadmill, particularly in terms of stance time. Other studies have also found that horse and human stance times were increased on treadmills (5, 7), possibly because the subject's body weight affects the speed control of the treadmill. Although the reported values for wheel running in Ref. 33 are for the hindlimb, they appear more similar to the forelimb values measured using our wheel, with the hindlimbs having longer stance times and higher duty factors. It is possible that this is because the wheel used had a fairly small diameter, meaning that the angle through which the paw could comfortably remain in contact was smaller than it would be on a flatter surface, and consequently, stance times were reduced.

### Automated Recording and Classification of Gaits Using the Wheel

Rather than attempting to place the gait samples into traditional gait categories, we seek to quantify the spectrum of gaits noted in Ref. 17 and suggested by the lack of “breakpoints” in our kinematic data. This quantity, 3S, is calculated based purely on the GRF traces and can be done automatically by computer. This quantity is calculated using the equation described in materials and methods.

Hildebrand (19) identifies a number of footfall timing measurements that are necessary and sufficient to describe gaits quantitatively; fore and hind stance times (typically presented as proportions of total stride time), difference in time between footfalls of the fore and hind pairs of legs (typically presented as a proportion of the appropriate stance time), difference in time between footfalls for ipsilateral fore and hind legs (typically presented as proportions of total stride time), and the sequence in which the legs of a pair contacts (typically presented as a proportion of the appropriate stance time). Gaits can also be described as “symmetric,” when the footfalls of the fore and hind leg pairs are spaced evenly in time (i.e., the time difference between footfalls is close to 50% of the stride time), or “asymmetric,” when the footfalls of a pair are not spaced evenly in time.

**Fig. 6.**
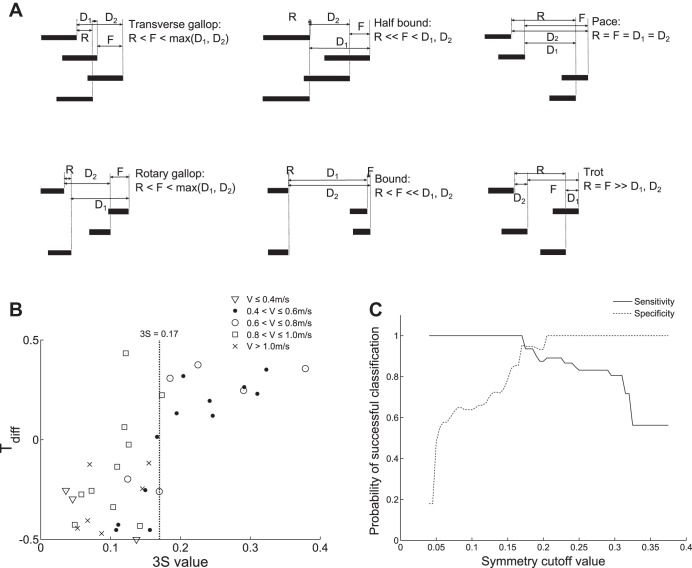
Comparison of 3S (stride signal symmetry) values and Hildebrand gait diagrams. *A*: example representative gait diagrams plotted using relative timings from Refs. 18 and 19. The phase difference between the fore feet is denoted as F, the phase difference between the hind feet is R, the phase differences between the diagonal pairs of legs are D_1_ and D_2_. *B*: plot of T_diff_ measured from the high-speed video vs. 3S, where T_diff_ = R − max(D_1_, D2). Markers denote the running speed, *V*, at the time of observation. The dotted line marks the boundary between asymmetric and symmetric gaits (in the 3S sense) at 0.17.

Figure 6*A* uses Hildebrand gait diagrams to illustrate a number of symmetric and asymmetric gaits. The phase differences between the fore, hind, and diagonal pairs of legs have been marked F, R, D1, and D2, respectively. Since symmetric gaits must have the footfalls of the fore and hind leg pairs out of phase, the smallest phase difference is seen between diagonal leg pairs. Conversely, since the footfalls of the fore and hind legs are not evenly spaced in asymmetric gaits they can be in phase (or close to in phase). This means that the phase difference between the fore and hind footfalls is less than the largest phase difference between the diagonal footfalls. In general, the phase difference between the hind legs is also less than between the fore legs.

Our gait classification algorithm also has a close relationship with footfall phase differences, in particular between the hind legs R, and between the two diagonal leg pairs D_1_ and D_2_. Figure 6*B* shows that there is a significant positive relationship (*r* = 0.73, *P* < 0.01) between 3S as calculated above, and T_diff_, where T_diff_ is defined as R − max(D_1_, D2) (normalized as a proportion of total stride duration). This corresponds to a 3S value of 0.17. We shall therefore refer to samples with 3S ≥ 0.17 as “symmetric” gaits and samples with 3S < 0.17 as “asymmetric” gaits.

Figure 6*C* plots specificity and sensitivity for the gait classification system. These two values express how well the algorithm is able to distinguish symmetric from asymmetric gaits; specificity measures the proportion of symmetric gaits that were correctly classified as symmetric, while sensitivity measures the proportion of asymmetric gaits that were correctly classified as not symmetric. The intersection of these two curves confirms that the optimum cut off between symmetric and asymmetric is 0.17, with a 95% probability of correctly classifying gaits at this threshold.

Overall, 193 traces (37.9%) were symmetric, while 316 traces (62.1%) were asymmetric. Conversely, in Ref. 17 it was found that 22.9% of the gaits were symmetric and 77.1% of the gaits were asymmetric. This could be because the speeds of wheel running reported here were selected by the mice, whereas the speeds of the treadmill in (17) were set by the experimenters, so the mice were able to choose speeds at which a symmetrical gait was preferable. This is corroborated by the fact that the majority (56.2%) of the gaits plotted (see Fig. 8) are between 0.4 and 0.7 m/s, the region where symmetric gaits are most common. The range of speeds of symmetric gaits was 0.08 to 1.0 m/s, and the range of speeds of the asymmetric gaits was 0.03 to 1.1 m/s. These results therefore agree with Ref. 17 that mice use both symmetric and asymmetric gaits throughout their speed range, with the majority of gaits being asymmetric.

With the use of the equations proposed in Refs. 1 and 16, a typical mouse with a hip height of 2.6 cm and a mass of 34 g should transition from walking to trotting between the speeds of 0.36 and 0.51 m/s and from trotting to galloping at a speed of 0.68 m/s. This corresponds to a walk-trot transition between the Froude numbers of 0.5 and 1 (calculated as *V*^2^/*gL* where *V* = speed, *g* = gravitational acceleration, and *L* = leg length; Ref. 2),and a trot-gallop transition at a Froude number of 1.8. In Ref. 34 a transition from trotting to a half bound was observed at a speed of 0.7 m/s. However, the authors of Ref. 17 could not identify any clear breakpoint in gaits and therefore suggested that rather than distinct gaits like many larger animals, mice display a “continuum of gaits.” This has a striking analogy with bipedal gaits, where smaller birds blur the distinction between walking and running gaits (14).

**Fig. 7.**
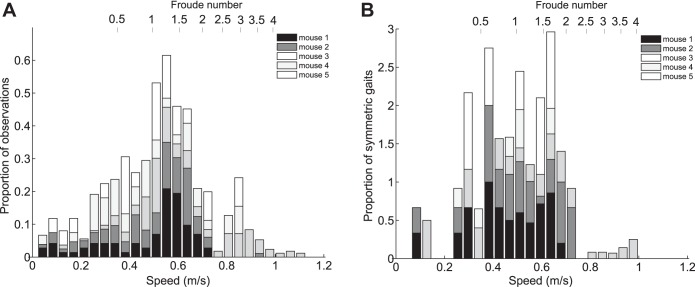
Histograms of speed and 3S for each mouse. *A*: overall frequencies of observations over the entire speed range. *B*: proportion of gait observations which were 3S symmetric vs. speed (relative to the total number of observations in each speed interval). Froude numbers (*V*^2^/*gL* where g = 9.81 m/s^2^ and *L* = 2.5 cm) are noted on the upper axis. The proportion of observed gaits that are 3S symmetric was highest between 0.29 and 0.69 m/s, and there are dips in frequency of observations at 0.38 ± 0.05 and 0.74 ± 0.04 m/s, suggesting that the “continuum of gaits” can be split into 3 regions: slow speed with a preference for 3S asymmetric (mostly creeping) gaits, medium speed with an equal division between 3S symmetric (trotting) and 3S asymmetric (mostly creeping or half bounding depending on speed) gaits, and high speed with a preference for 3S asymmetric (galloping and half bounding) gaits.

In Fig. 7*A* speed is plotted against the frequency of observations at each speed for each mouse. All five mice covered a similar speed range from 0.06 ± 0.04 to 0.90 ± 0.13 m/s. In all cases most of the observations were at speeds around the middle of the speed range, with a mean preferred speed of 0.50 ± 0.13 m/s (1.99 ± 0.52 rps), with the number of observations dropping off as speed increased or decreased. This agrees with Ref. 12, which found that mice spent most of their time running at a “cruising speed” of 2.5 rps. However, unlike Ref. 12 the frequency of observations increases before and after two minima at 0.38 ± 0.05 and 0.74 ± 0.04 m/s, resulting in two additional possible “preferred speeds” at 0.33 ± 0.04 and 0.88 ± 0.04 m/s (although these speeds are still not used as much as the peak preferred speed). These could correspond to the preferred speeds for different gaits (21).

Most cursorial quadrupeds use trotting or low speed gallops when traversing long distances (6); these gaits tend to fall in the middle of an animal's speed range. Similarly, ground squirrels in the wild run between feeding and nesting sites at speeds close to or slightly above the middle of their speed range; not only does this minimize their exposure to predators, it reduces the net energy cost by 73% compared with walking the same distance (26). While wheel running differs from these examples in that the animal is not attempting to reach a particular location in a limited amount of time, it is possible that wheel use, as a relatively long-term, nonexploratory mode of locomotion, may be subject to similar biomechanical constraints. Additionally, both mice (27) and ground squirrels (22) are able to increase their running speed with relatively little increase in rate of energy expenditure. Running could therefore be preferable to walking, especially if the mouse is attempting to maximize the distance it has “travelled.” It is also possible that the inertia of the wheel makes continuous walking difficult, and therefore, the mouse runs instead. In both cases, the preferred speed may be one that minimizes energetic cost for the selected gait (21); typically this is near the middle of the speed range for that particular gait, rather than at the minimum or maximum speeds.

**Fig. 8.**
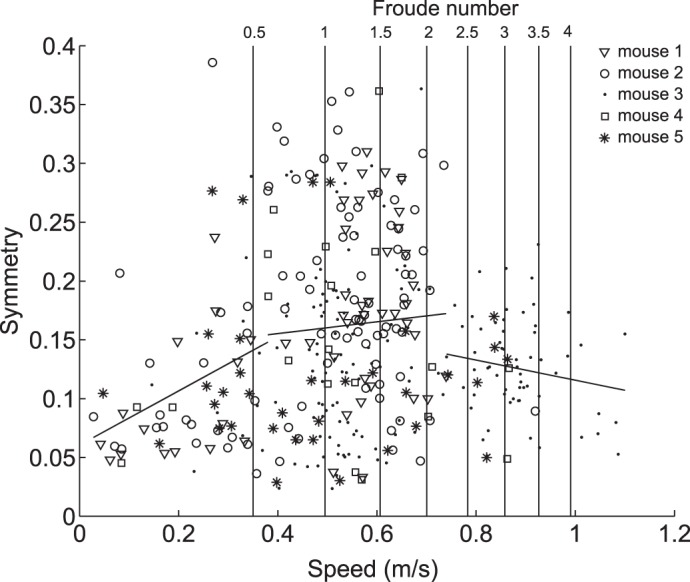
3S values of gaits vs. speed. Markers denote which mouse was running; all mice used a range of both 3S symmetric and 3S asymmetric gaits throughout their speed range. 3S significantly increased with speed at speeds below 0.38 m/s (*y* = 0.06 + 0.23*x*, *r* = 0.32, *P* = 0.009), increased at a slower rate between 0.38 and 0.74 m/s (*y* = 0.14 + 0.05*x*, *r* = 0.05, *P* = 0.43) and decreased with speed at speeds above 0.74 m/s (*y* = 0.20 − 0.09*x*, *r* = −0.17, *P* = 0.18).

In Fig. 7*B* speed is plotted against frequency of 3S symmetric gaits as proportions of the total number of traces in each speed bin; the majority of symmetric gaits occur between the speeds 0.29 ± 0.04 and 0.69 ± 0.06 m/s. These results suggest that while the mice used both 3S symmetric and 3S asymmetric gaits throughout their speed range, this “continuum of gaits” can be divided into three distinct regions defined by the frequency of 3S symmetric and 3S asymmetric gaits at each speed. Hoyt and Taylor (21) found that, when allowed to self-select its speed, a horse ran at speeds that minimized metabolic cost and avoided using speeds close to the transition speeds between gaits. Similarly, the reductions in observations around 0.38 and 0.74 m/s suggest that these divisions in the continuum of gaits are analogous to the gait transitions observed in larger animals such as horses. These speeds are also close to the predicted gait transition speeds for mice of 0.36–0.51 and 0.68 m/s. If the continuum of gaits is split at these points, relationships between speed and 3S values can be observed (see Fig. 8): below 0.38 m/s there is a significant positive relationship between 3S and speed (*r* = 0.24, *P* = 0.033), between 0.38 and 0.74 m/s there is no significant relationship between 3S and speed (*r* = −0.006, *P* = 0.91), and above 0.74 m/s there is a negative relationship between 3S and speed (*r* = −0.18, *P* = 0.054). The boundaries between these regions are similar to the values predicted from the equations in Refs. 1 and 16, suggesting that scaling relationships based on dynamic similarity do hold true for mice and that speed does have some influence on mouse gaits. However, the variability of gaits suggests that this relationship is less strict than in larger animals. Studies of locomotion energetics in other small rodents have found that cost of transport remained constant (24) or even decreased with speed (22), suggesting that energy is less important in gait transitions than for larger animals (21). Iriarte-Díaz et al. (24) also found that increased body mass did not change trot-gallop transition speed in degus, unlike horses, suggesting that mechanical stresses also play less of a role in gait transitions for smaller animals. If the “fitness function” determining energetic and mechanical costs has a flatter landscape for smaller animals, this could explain why mice are able to use both symmetric and asymmetric gaits throughout their speed range.

### Conclusions

#### Wheel implementation.

The wheel presented here fills a niche for a noninvasive, automated system for monitoring rodent activity and locomotor biomechanics. Unlike conventional force plates or treadmills the wheel can be installed in a cage and mice will use it voluntarily, with no human supervision or interaction required. Furthermore, apart from an acclimatization period, animals do not need to be trained to use the wheel. Since the wheel is made up of multiple force pads data can be collected for multiple legs throughout locomotion, rather than just for a single foot as in Ref. 33, making it possible for different gaits to be identified and analyzed. Additionally, the use of 3D printing to manufacture the wheel means that the design can be shared on the internet for the use of other research institutes and can be easily deployed in multiple cages in parallel

The system presented in this paper takes advantage of the motivation of mice to run on exercise wheels; it is therefore most useful for capturing large amounts of data (or data over a long time period) relating to voluntary locomotion. However, in some situations other setups may be more appropriate; for example, if a researcher wishes to select the speed or duration of running bouts, a treadmill would provide more control. Similarly, although force magnitudes can be measured, the wheel has a tendency to underestimate contact times at low speeds, making stance and duty factor measurements less accurate. This is most likely due to the fact that the lower forces at these speeds (particularly at the beginning and end of stance) fall below the sensitivity of the wheel. It might be possible to improve sensitivity, for example, by using thinner meandered elements to reduce their stiffness; however, this would also impact the wheel's robustness under continual use. Additionally, since it is not possible to determine where each foot contacts a pad, center of pressure cannot be determined from the wheel, so if it is desirable to calculate internal joint angles, a more sensitive fixed force platform may be preferable.

#### Automated recording and classification of gaits using the wheel.

The wheel was used to investigate how mouse gaits change with speed, using a metric called 3S; gaits with 3S values greater or equal to 0.17 were found to correspond well with symmetric gaits as defined by Hildebrand, while gaits with 3S values less than 0.17 corresponded with asymmetric gaits. It was found that although mice can use both symmetric and asymmetric gaits throughout their entire speed range, the continuum of gaits can be split into three regions. At low speeds, the mice mostly used asymmetric gaits; video data for gaits in this range identified these as creeping. As speed increased, symmetric gaits became more common, until the first boundary where approximately half the gaits were symmetric and half were asymmetric. Symmetric gaits in this region were identified as trotting, and asymmetric gaits were identified as half bounding or galloping. This continued until the second boundary, after which the majority of gaits were asymmetric, and 3S values decreased as speed increased. Gaits were identified from the video as being half bounding or galloping. These data suggest that mouse gaits are dependent on speed, although to a lesser extent than some larger animals.

One goal of this algorithm was to allow gait analysis without requiring high-speed video; video storage and processing can be expensive in terms of time and computational capability, and setting up cameras to continually monitor activity over long time periods can be difficult, especially in the home cage environment where mice may move bedding in front of the camera.

To some extent this has been achieved; it is possible to distinguish symmetric and asymmetric gaits throughout the speed range of the mice and to measure changes in kinematic parameters with speed. However, there are still instances when having access to camera data may be advantageous: the algorithm alone cannot distinguish whether a lead leg is a right or a left leg, and it cannot distinguish between similar gaits such as rotary and transverse gallops, or diagonal and lateral walks. A camera would also be useful for identifying anomalous results caused by multiple mice running at once or a mouse “coasting” rather than running. There are also limitations to analyzing gaits based on wheel running, although it appears wheel running results in more similar kinematics to overground running than treadmill running. Mice do not tend to walk consistently on wheels, making comparative studies of low speed locomotion difficult. The inertia and curved shape of the wheel may also have some effect on stance times and duty factors.

In spite of these limitations, we believe that this wheel can be a useful tool for mouse gait analysis, particularly in situations where large datasets can be collected and the impact of anomalous signals would be reduced. The wheel could also be used in conjunction with a camera to identify activity periods of interest, reducing the need to go through hours of video to find events.

The gait classification algorithm offers a way to describe gaits that do not fit into the usual discrete gait categories; mice (in common with many other small mammals and birds) do not have clearly identifiable break points when kinematic parameters are plotted against speed, they also use much more “grounded” gaits with higher duty factors than larger animals moving at dynamically similar speeds. This could suggest that smaller animals do not have as strict a relationship between speed and gait as larger animals and may not transition between gaits due to the same biomechanical factors. Attempting to classify small animal gaits in the same way as large animal gaits may therefore be dividing them into artificial categories that do not necessarily correspond to biomechanical state-spaces. Instead, our quantitative gait metric aims to provide a continuous scale on which two gaits with similar speeds can have very different kinematics. By capturing these distinctions, it may be easier to identify what physical and physiological triggers lie behind gait selection in mice and other small animals.

## GRANTS

This work was funded by Wellcome Trust Fellowship 095061/Z/10/Z (to J. R. Usherwood). The RVC manuscript number of this paper is CBS_00895.

## DISCLOSURES

No conflicts of interest, financial or otherwise, are declared by the author(s).

## AUTHOR CONTRIBUTIONS

Author contributions: B.J.S. conception and design of research; B.J.S. and L.C. performed experiments; B.J.S. and L.C. analyzed data; B.J.S. and J.R.U. interpreted results of experiments; B.J.S. prepared figures; B.J.S. drafted manuscript; B.J.S., L.C., and J.R.U. edited and revised manuscript; B.J.S., L.C., and J.R.U. approved final version of manuscript.

## Figures and Tables

**Fig. A1.**
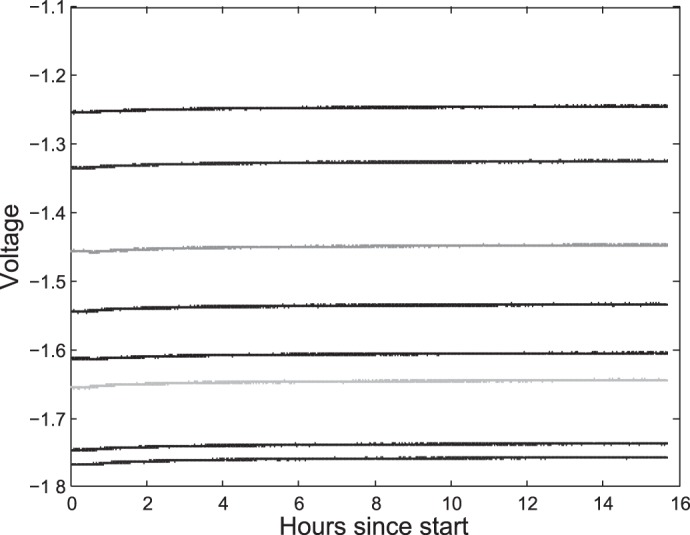
Drift over time of the wheel sensors. Over a period of more than 15 h there was less than 1% drift in any of the sensors.

**Fig. B1.**
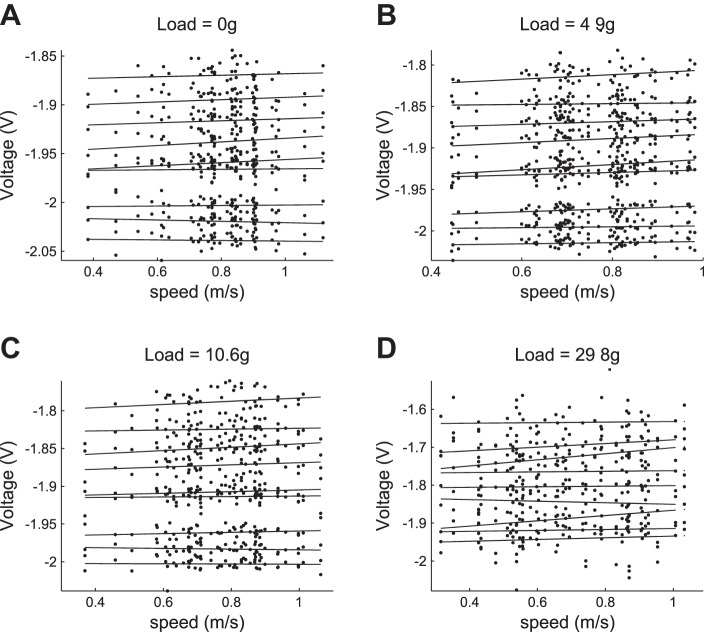
Effect of speed on sensor voltages at different loads. Although in some cases there are significant effects of speed, there appears to be no systematic relationship between speed and voltage.

## Appendix A: DRIFT IN SENSORS OVER TIME

Figure A1 shows drift over time of the wheel sensors. Table A1 shows drift values over time expressed in volts and as a percentage of the starting value.

**Table A1.** Drift values over time expressed in volts and as a percentage of the starting value

Sensor	Drift, mV	Drift, %
0	−11.5	−0.75
1	−11.2	−0.64
2	−10.2	−0.58
3	−8.20	−0.57
4	−8.20	−0.62
5	−10.9	−0.66
6	−7.90	−0.49
7	−6.90	−0.55
8	−9.90	−0.64

In all cases drift is <1% of the starting value.

## Appendix B: EFFECT OF SPEED ON SENSOR VOLTAGES

Figure B1 shows the effect of speed on sensor voltages at different loads. Table B1 shows correlation coefficients and *P* values for speed vs. sensor voltage.

**Table B1.** Correlation coefficients and P values for speed vs. sensor voltage

Sensor	Unloaded	4.9 g	10.6 g	29.8 g
1	*r* = 0.21, *P* = 0.15	*r* = 0.25, *P* = 0.045*	*r* = 0.16, *P* = 0.25	*r* = 0.02, *P* = 0.88
2	*r* = 0.08, *P* = 0.60	*r* = 0.24, *P* = 0.057	*r* = 0.18, *P* = 0.21	*r* = 0.03, *P* = 0.85
3	*r* = 0.24, *P* = 0.099	*r* = 0.36, *P* = 0.003*	*r* = 0.14, *P* = 0.33	*r* = 0.04, *P* = 0.82
4	*r* = 0.13, *P* = 0.38	*r* = 0.20, *P* = 0.10	*r* = 0.28, *P* = 0.041*	*r* = 0.37, *P* = 0.019*
5	*r* = 0.15, *P* = 0.32	*r* = 0.07, *P* = 0.60	*r* = 0.08, *P* = 0.57	*r* = 0.22, *P* = 0.17
6	*r* = 0.04, *P* = 0.79	*r* = 0.25, *P* = 0.042*	*r* = 0.05, *P* = 0.72	*r* = −0.07, *P* = 0.65
7	*r* = 0.05, *P* = 0.73	*r* = 0.31, *P* = 0.012*	*r* = 0.16, *P* = 0.26	*r* = 0.32, *P* = 0.039
8	*r* = −0.06, *P* = 0.69	*r* = 0.14, *P* = 0.27	*r* = −0.03, *P* = 0.85	*r* = 0.13, *P* = 0.40
9	*r* = −0.16, *P* = 0.29	*r* = 0.10, *P* = 0.44	*r* = −0.09, *P* = 0.51	*r* = 0.07, *P* = 0.65

* Statistical significance at *P* < 0.05.
